# Decoding JFT: a multifunctional fluorescence probe for sulfite and viscosity insights

**DOI:** 10.3389/fchem.2025.1642191

**Published:** 2025-07-23

**Authors:** Hua Han, Bin Han, Yongjin Peng, Yuling Liu

**Affiliations:** ^1^ 3rd Affiliated Hospital of Jinzhou Medical University, Jinzhou, China; ^2^Department of Physics, Tianjin Renai College, Tianjin, China; ^3^College of Modern Industry of Health Management, Jinzhou Medical University, Jinzhou, China; ^4^Liaoning Province Key Laboratory of Human Phenome Research, Jinzhou Medical University, Jinzhou, China

**Keywords:** multifunctional, fluorescent probe, sulfite, intracellular viscosity, theoretical investigation

## Abstract

This study focuses on a multifunctional fluorescence probe JFT based on the FRET (Fluorescence Resonance Energy Transfer) and TICT (Twisted Intramolecular Charge Transfer) mechanism. JFT combines an electron donor and an acceptor, enabling it to detect sulfite and monitor intracellular viscosity. When reacting with sulfite, its electronic structure changes, turning off FRET and altering fluorescence wavelength and intensity. In different viscosity environments, the rotation of carbon-carbon bonds in the electron acceptor structure of JFT varies, affecting non-radiative transition pathways and fluorescence intensity. Theoretical calculations based on TDDFT reveal the electron distribution changes before and after the reaction with sulfite species, consistent with experimental phenomena. These findings deepen the understanding of the FRET mechanism of fluorescence probes and offer theoretical guidance for the design of more efficient fluorescence probes.

## Introduction

Fluorescence probes have emerged as powerful tools in biological and chemical research, enabling real-time and non-invasive monitoring of various analytes and environmental parameters (Peng et al., 2024; Liu et al., 2024a; Liu et al., 2024b; Sun et al., 2019; Liu et al., 2024c). In the context of intracellular studies, simultaneously monitoring the changes in intracellular sulfite concentration and viscosity is of great significance. Sulfite is an important signaling molecule in the body, involved in many physiological and pathological processes, such as vasodilation, neurotransmission, and antioxidant defense (Zhang W. J. et al., 2022; Liu et al., 2023; Liu et al., 2022; Wang et al., 2024; Ren et al., 2024; Li et al., 2023). Abnormal levels of sulfite are associated with various diseases, including cardiovascular diseases, neurodegenerative diseases, and respiratory diseases. Therefore, accurately detecting the concentration of sulfite in cells is crucial for understanding its biological functions and related pathological mechanisms.

On the other hand, intracellular viscosity is a key physical parameter that reflects the micro-environment of cells. It affects many cellular processes, such as protein folding, enzyme activity, membrane fluidity, and intracellular transport. Changes in intracellular viscosity are often related to cell proliferation, differentiation, and apoptosis. Thus, monitoring intracellular viscosity can provide valuable information about cell status and function (Peng et al., 2024; Liu et al., 2024c; Ma et al., 2024; Zhang C. et al., 2022; Arachchige et al., 2024; Wang et al., 2021; Peng et al., 2023).

However, developing a fluorescence probe that can simultaneously monitor these two parameters is challenging. Most traditional fluorescence probes are designed to detect only one analyte. The development of a multifunctional probe requires a careful combination of different sensing mechanisms.

Recently, Wang et al. developed a multifunctional fluorescence probe JFT based on the FRET/TICT mechanism (Wang et al., 2025). This probe effectively combined an electron donor and an acceptor, endowing it with unique properties for detecting sulfite and monitoring viscosity. The FRET property of the probe allowed for sensitive detection of sulfite through changes in fluorescence emission wavelength and intensity. Meanwhile, the rotation of carbon-carbon bonds in the electron acceptor structure of the probe was sensitive to environmental viscosity, enabling viscosity-dependent fluorescence changes. This paper aimed to comprehensively explore the working mechanism of the JFT fluorescence probe, including its response to sulfite and viscosity changes, as well as the underlying structural and electronic changes, which was expected to provide in-depth insights into fluorescence probe design and expand its applications in biological and chemical analysis.

## Theoretical methods

The geometric optimization and vibrational frequency analysis on the probe JFT were carried out at the PBE0/def2-TZVPD level with D3 dispersion and GCP correction to remove artificial overbinding effects from BSSE using ORCA 5.0.1 program (Neese, 2022; Su et al., 2013; Laun and Bredow, 2022). To explore the excited-state properties of the molecule, the time-dependent density functional theory (TDDFT) computations were conducted at the CAM-B3LYP/def2-TZVPD level (Shao et al., 2020). The charges of the probe JFT and its reaction product with sulfite were +1 and 0 respectively. DMSO environment under the SMD solvent model was used in calculation which was align with the experimental work. All molecular visualizations were generated via the VMD 1.9.3 software, and the in-depth electronic structure analyses were accomplished by employing the Multiwfn 3.8 (dev) package (Lu and Chen, 2012; Humphrey et al., 1996).

## Results and discussion

**SCHEME 1:**
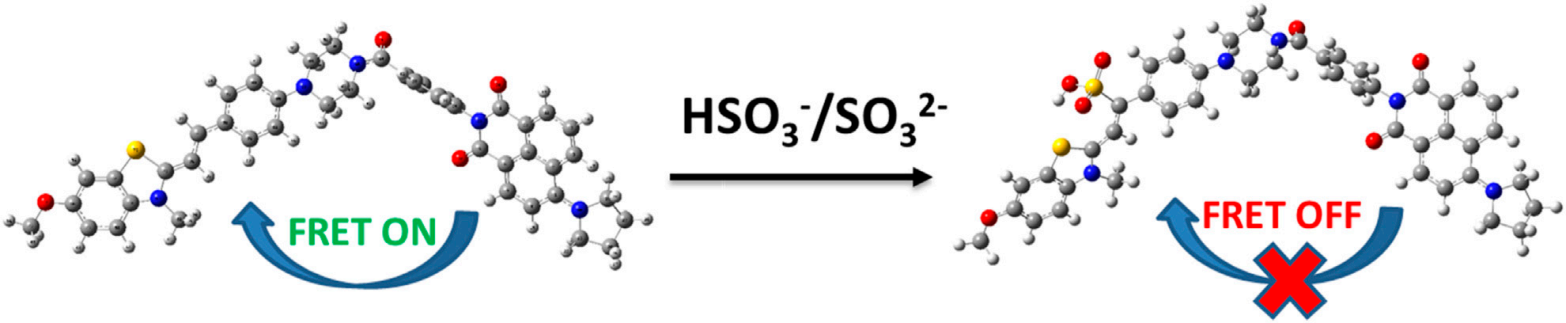
The sensing mechanism of JFT toward HSO_3_^−^/SO_3_^2−^.

The emission spectrum of the electron donor of probe JFT could well overlap with the absorption spectrum of the acceptor of it, a characteristic that endowed the probe JFT with FRET properties. After the probe reacted with sulfite, the change in its electronic structure could turn off the FRET of probe JFT, thus causing significant changes in its fluorescence emission wavelength and intensity, making it an excellent sulfite detection probe. This mechanism was shown in Scheme 1.

Meanwhile, the electron acceptor structure in probe JFT contained C-C bonds with a high degree of rotational freedom. In a low-viscosity environment, these carbon-carbon bonds could rotate relatively freely, transferring energy among different conformations. This provided some non-radiative transition pathways for probe JFT to de-excite from the excited state to the ground state. When the environmental viscosity increased, the rotation of these carbon-carbon bonds was inhibited, and the non-radiative transition pathways for the probe to de-excite from the excited state to the ground state were cut off, thereby enhancing the corresponding fluorescence intensity. This enabled probe JFT to effectively monitor the intracellular viscosity level.

**FIGURE 1:**
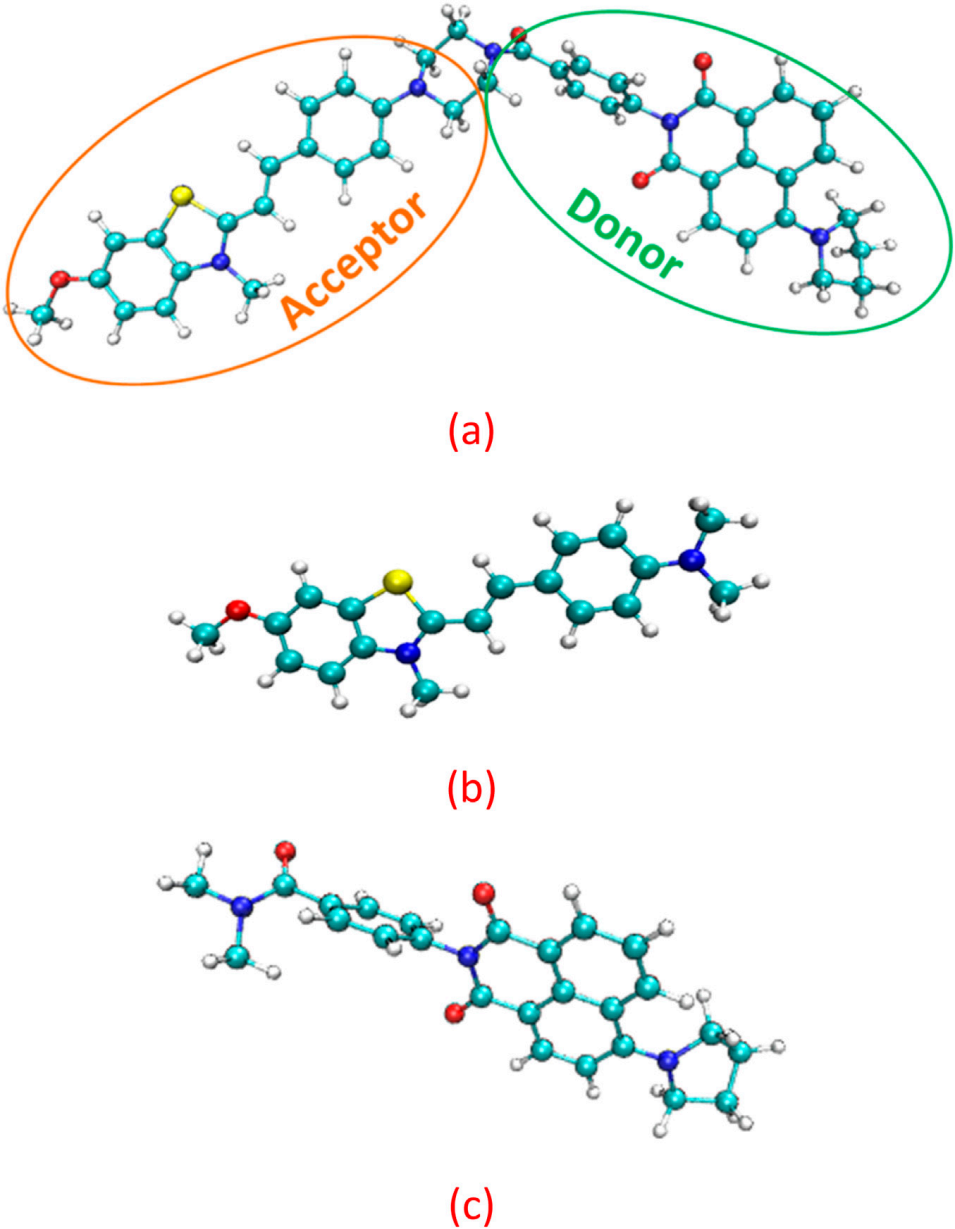
The structure of the **(a)** fluorescent probe JFT **(b)** acceptor **(c)** donor.

**FIGURE 2:**
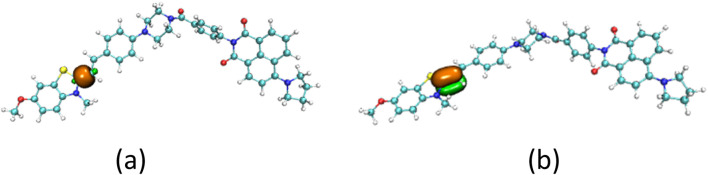
The σ component **(a)** and π component **(b)** of the c-c bond in probe JFT.

The structures of the JFT fluorescent probe and its electron donor and acceptor parts (same as Scheme 2 in Wang et al. (2025)) were shown in Figure 1. From the structural diagram of the electron acceptor part, it could be seen that the carbon-carbon bonds connecting the N-methylbenzothiazole and dimethylaniline had a large degree of rotational freedom, and the carbon-carbon double bond was also the reaction site with HSO_3_^−^/SO_3_^2−^. To study the rotational freedom of these carbon-carbon bonds, the compositional characteristics of the carbon-carbon bond in the probe JFT were analyzed through natural adaptive orbital concept and shown in Figure 2. The carbon-carbon bond had a relatively large proportion of easily rotatable σ component (70%), while the non-easily rotatable π component accounted for a relatively small proportion (28%).

**FIGURE 3:**
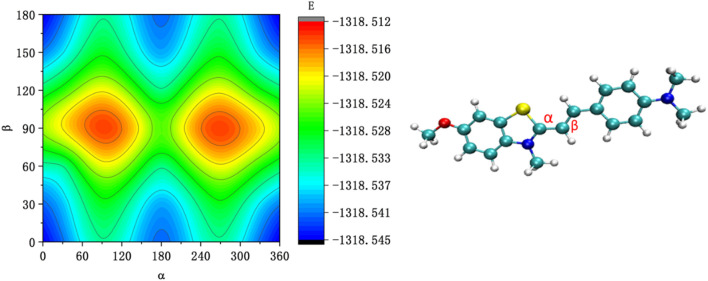
The energy scan of the JFT probe’s acceptor based on the rotation of α and β carbon-carbon bonds.

To further study the conformational changes of the JFT fluorescence probe brought about by the rotation of these carbon-carbon bonds, the energy scan of the JFT probe’s acceptor based on the rotation of these carbon-carbon bonds was carried out (Figure 3). It can be seen from Figure 3 that when N-methylbenzothiazole and dimethylaniline were in the same plane, the probe was in the lowest-energy conformation. When the carbon-carbon bond rotated to other angles, the probe was in other local low-energy conformations. The energy barriers between these conformations were relatively low. Therefore, in a low-viscosity environment, conversions between different conformations could occur relatively easily, providing a non-radiative energy dissipation path for the de-excitation of the JFT fluorescence probe, resulting in a weak fluorescence intensity of the JFT probe in this state. When the environmental viscosity increased, the rotation of the carbon-carbon bonds in the probe was inhibited. When the JFT probe de-excited from the excited state back to the ground state, the fluorescence radiation path would be highly prioritized, thus causing a significant increase in fluorescence intensity. This conclusion was consistent with the experimental phenomena.

**FIGURE 4:**
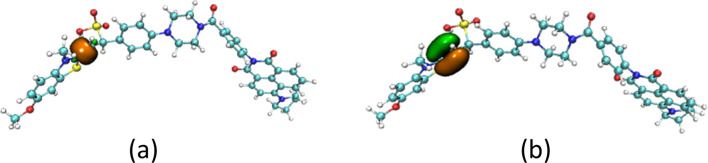
The σ component **(a)** and π component **(b)** of the c-c bond in product of JFT probe reacted with HSO_3_^−^.

Another way to inhibit the rotation of the carbon-carbon bonds in probe JFT was that the composition of the carbon-carbon bonds was changed after the probe reacted with HSO_3_^−^/SO_3_^2−^. The proportion of rotatable σ component versus non-rotatableπ component in the carbon-carbon bond changed from 70%:28%–57%:42% as shown in Figure 4. The addition of the HSO_3_^−^ group significantly increased the inhibitory effect on the rotation of the carbon-carbon bonds, thereby also causing an enhancement in the fluorescence intensity of probe JFT under optical excitation.

**FIGURE 5:**
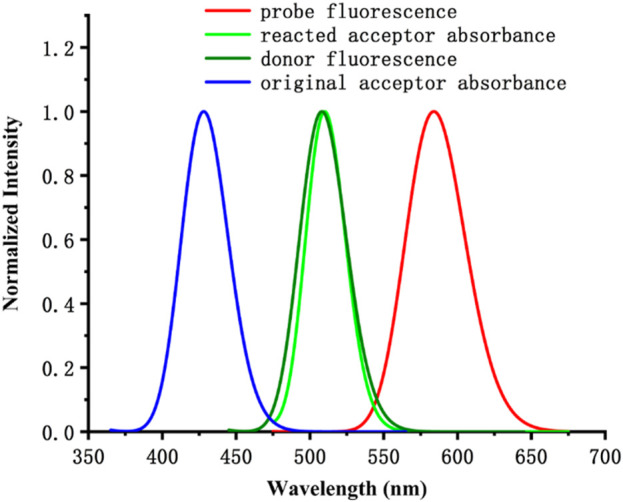
The calculated absorbance and fluorescence spectrum of the probe and its donor/acceptor.

At the same time, the introduction of the HSO_3_^−^ group changed the electronic structure of the electron acceptor in probe JFT, so that the absorption spectrum of the changed acceptor no longer overlapped with the emission spectrum of its donor, thus cutting off the original FRET channel of the probe, as shown in Figure 5. This structural change caused significant changes in the fluorescence emission wavelength and intensity of probe JFT. The intensity of the original orange fluorescence (582 nm) was significantly weakened, while the intensity of the green fluorescence (530 nm) was significantly enhanced. Experimental results showed that there was a good linear relationship between the ratio I_530_/I_582_ and the concentration of HSO_3_^−^/SO_3_^2−^ in the environment. Therefore, the fluorescence changed value I_530_/I_582_ of probe JFT became an effective indicator for monitoring the concentration of HSO_3_^−^/SO_3_^2−^ in the environment.

To further reveal the influence of the electronic structure changes of probe JFT before and after the reaction with HSO_3_^−^/SO_3_^2−^ on its fluorescence emission, the electron distribution of probe JFT in the ground state and excited state was studied based on the TDDFT method.

**FIGURE 6:**
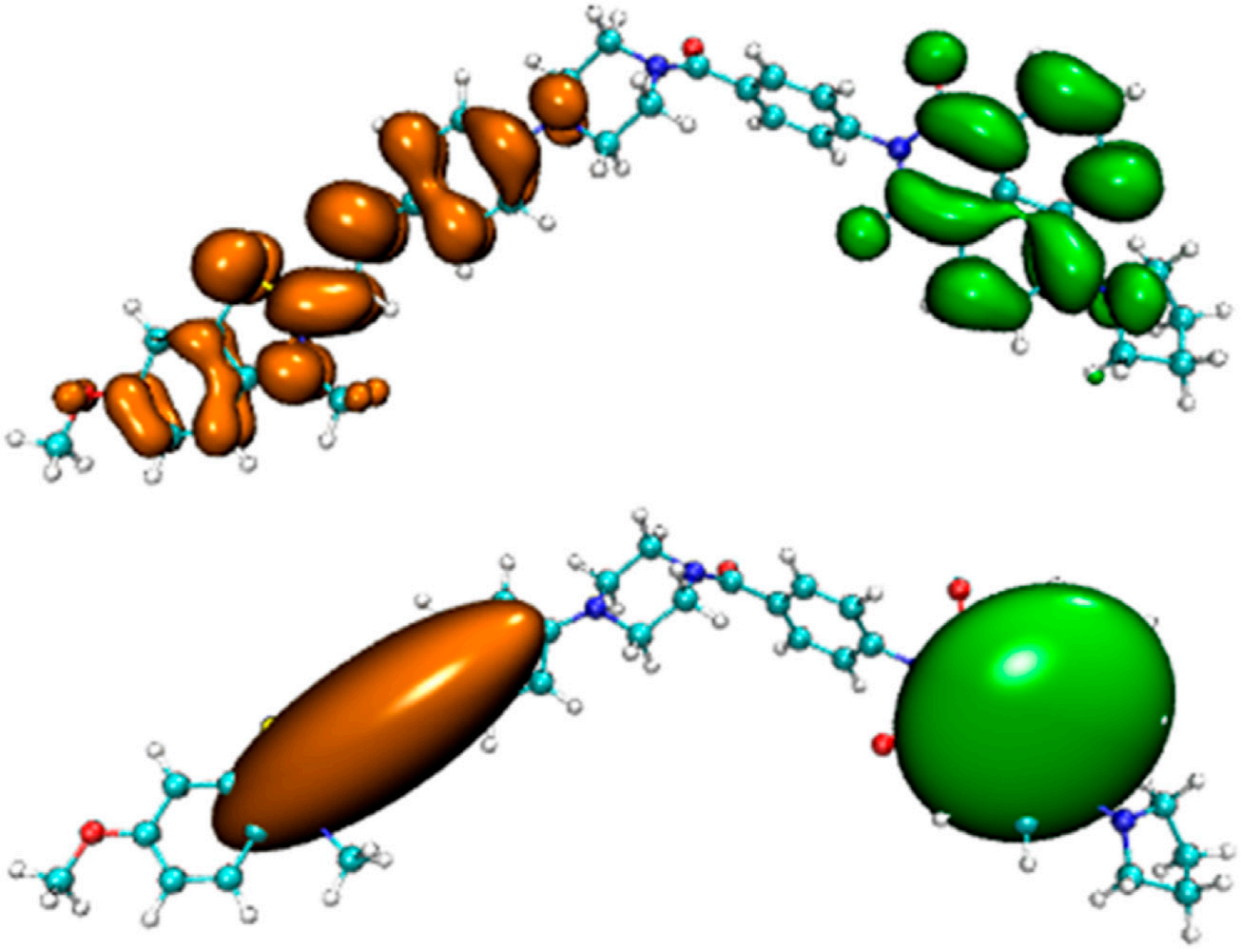
Difference diagram of electron distribution between S_0_ and S_1_ of probe JFT (orange:hole, green:electron).

**FIGURE 7:**
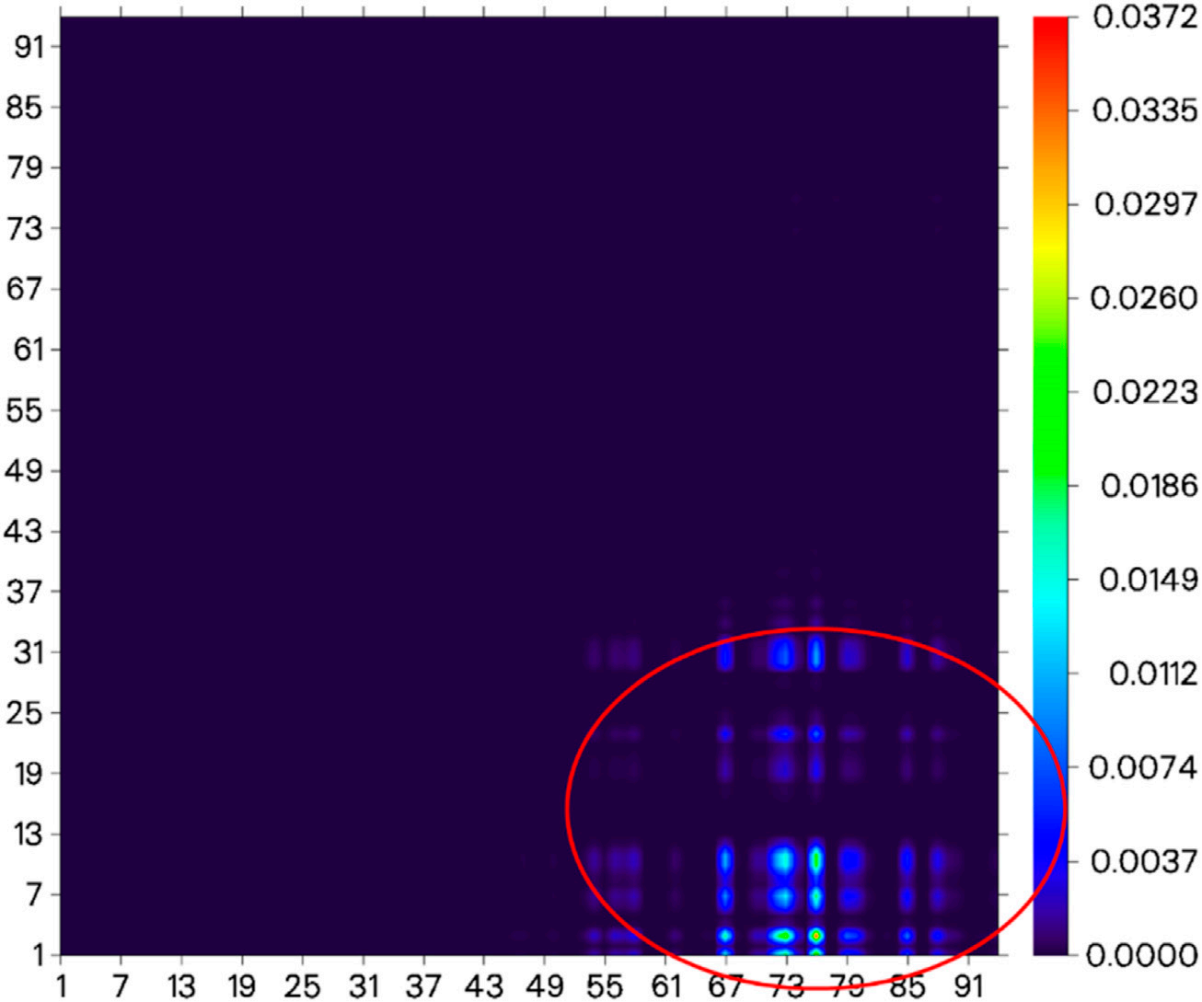
Electron-transfer heat map between S_0_ and S_1_ of probe JFT (donor part:1–31 to acceptor part:67–91 as shown in the red circle, the atom number list could be referenced to Supplementary Figures S1, S2).

**FIGURE 8:**
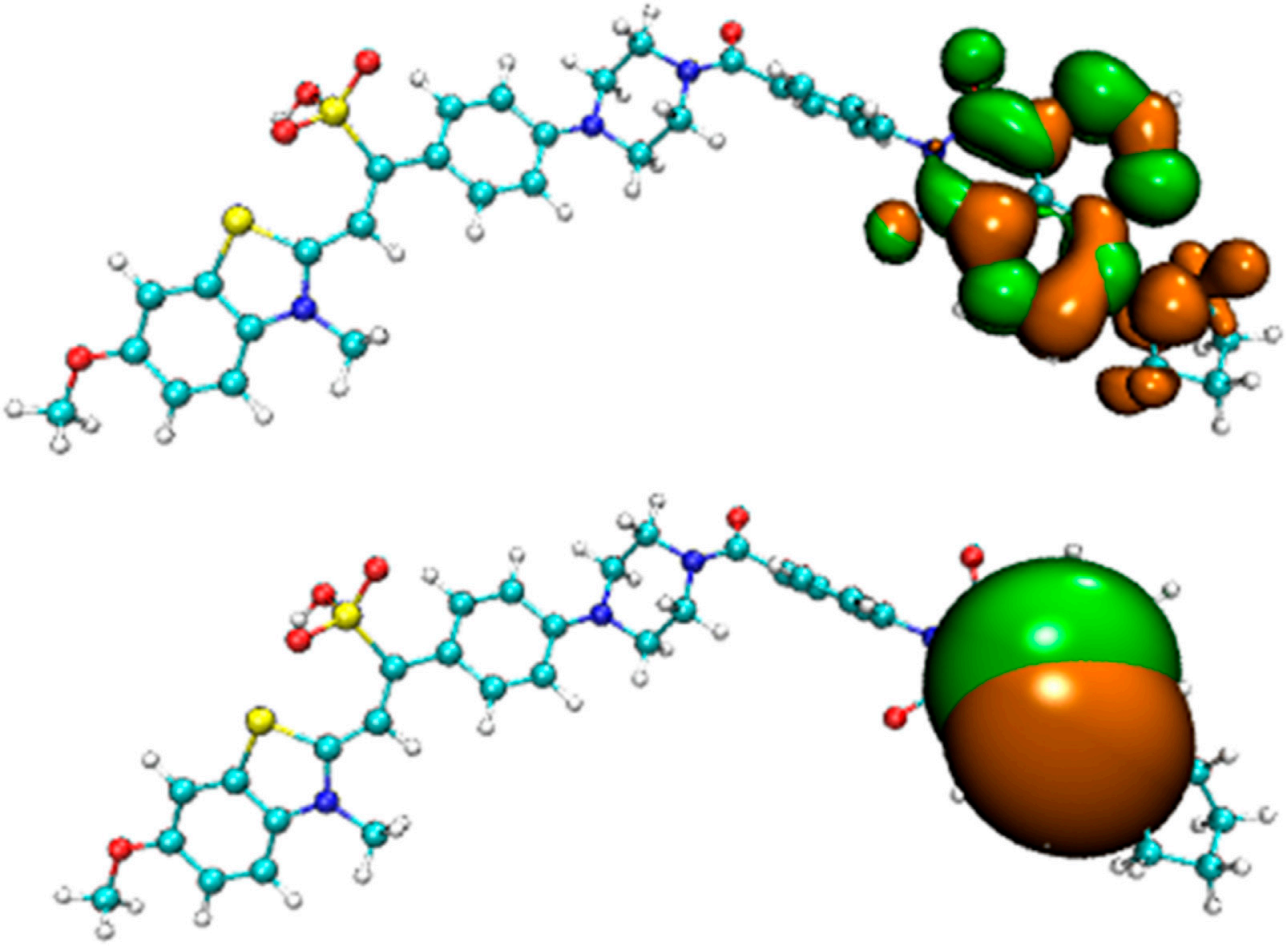
Difference diagram of electron distribution between S_0_ and S_1_ of the product of probe JFT reacted with HSO_3_^−^ (orange:hole, green:electron).

**FIGURE 9:**
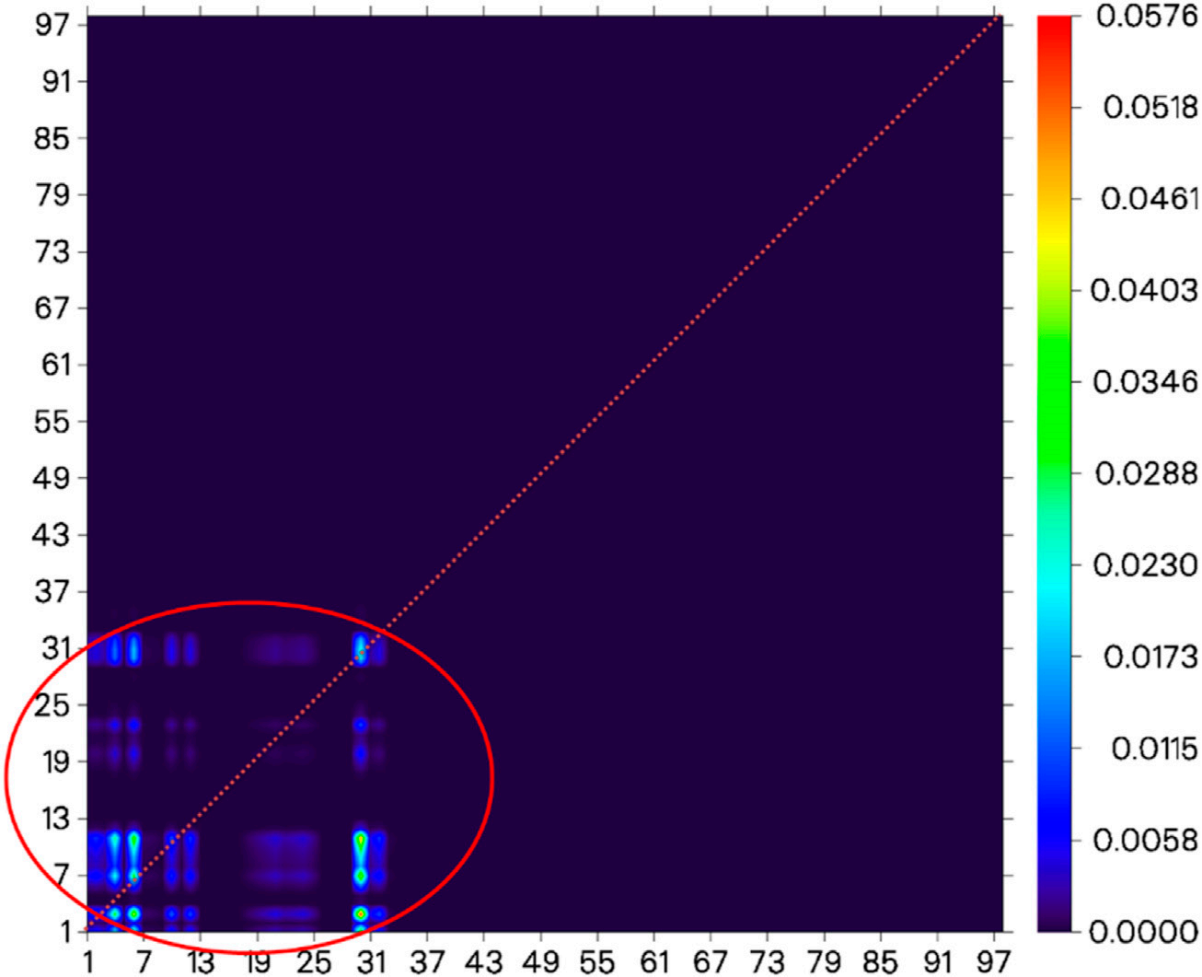
Electron-transfer heat map between S_0_ and S_1_ of the product of probe JFT reacted with HSO_3_^−^ (donor part:1–31 to donor part:1–31 as shown in the red circle, the atom number list could be referenced to Supplementary Figures S1, S2).

Before the reaction with HSO_3_^−^/SO_3_^2−^, the difference in electron distribution between the ground state S_0_ and the first excited state S_1_ of probe JFT showed obvious charge-transfer characteristics, which was consistent with the calculation results of the emission and absorption spectra of the probe’s electron donor and acceptor as mentioned before. These characteristics could be clearly seen from the difference diagram of electron distribution between S_0_ and S_1_ as shown in Figure 6, and which atoms participate in the charge-transfer process could be further obtained from the electron-transfer heat map generated through Multiwfn program as shown in Figure 7. When probe JFT reacted with HSO_3_^−^/SO_3_^2−^, due to the change in the geometry and electronic structure of the electron acceptor, the FRET process was interrupted. The difference diagram of electron distribution between S_0_ and S_1_ of the product as shown in Figure 8 clearly showed the local excitation characteristics between the two states at this time, and the fluorescence color changed from orange (582 nm) to green (530 nm). Although due to the limitation of accuracy, there was some deviation within the calculated wavelength value from the experimental measurement value, the amplitude and trend of the wavelength change were in good agreement with the experiment. The difference diagrams of electron distribution between S_0_ and S_1_ and the electron-transfer heat map (Figure 9) both showed that electron excitation only occurred in the electron donor part of the probe at this time. The calculated results of the fluorescence emission wavelength were consistent with the experiment. The absorption and emission characteristic values of probe JFT before and after the reaction with HSO_3_^−^/SO_3_^2−^ and the corresponding donor and acceptor structure () were shown in Tables 1, 2 respectively. These theoretical results deepened our understanding of the FRET mechanism of fluorescence probes and provided theoretical inspiration for designing more efficient fluorescence probes based on these mechanisms in the future.

**TABLE 1:** The main electron excitation processes in the JFT probe and its product reacting with HSO_3_^−^/SO_3_^2−^.

Probe	Electronic transition^a^	Excitation energy	Oscillator strengh	Composition^b^	CI^c^
JFT	S_0_ 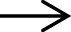 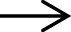 S_1_	408 nm	2.1804	H 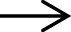 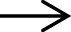 L	0.7105
Donor	S_0_ 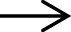 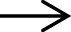 S_1_	403 nm	2.0042	H 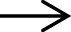 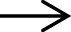 L	0.6892
Acceptor	S_0_ 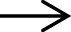 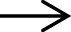 S_1_	426 nm	2.1465	H 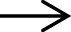 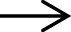 L	0.6819
Product	S_0_ 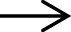 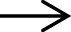 S_1_	514 nm	2.1864	H 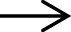 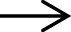 L	0.7014

^a^ Only the excited states with oscillator strength larger than 0.1 were considered.

^b^ H stand for HOMO and L stands for LUMO.

^c^ Coefficient of the wave function for each excitation was in absolute value.

**TABLE 2:** The main emission processes in the JFT probe and its product reacting with HSO_3_^−^/SO_3_^2−^.

Probe	Electronic transition^a^	Emission energy	Oscillator strengh	Composition^b^	CI^c^
JFT	S_1_ 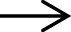 S_0_	578 nm	2.2046	L 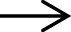 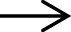 H	0.7305
Donor	S_1_ 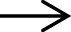 S_0_	503 nm	2.1121	L 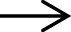 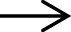 H	0.6372
Acceptor	S_1_ 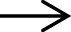 S_0_	561 nm	2.0437	L 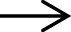 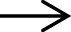 H	0.6789
Product	S_1_ 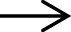 S_0_	524 nm	2.1504	L 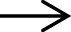 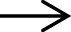 H	0.6914

a, b, c same indication as in Table 1.

## Conclusion

The development of the multifunctional fluorescence probe JFT represented a significant advancement in the field of fluorescence detection. Its ability to simultaneously monitor intracellular sulfite concentration and viscosity provided a powerful tool for studying related physiological processes. The combination of the FRET/TICT mechanism allowed the probe to respond sensitively to changes in sulfite levels and environmental viscosity. The clear understanding of the role of carbon-carbon bond rotation in fluorescence intensity regulation, as well as the influence of HSO_3_^−^/SO_3_^2−^ on the electronic structure and FRET process, provided a solid theoretical basis for the probe’s performance. The good linear relationship between the fluorescence change value I_530_/I_582_ and HSO_3_^−^/SO_3_^2−^ concentration demonstrated the probe’s potential for quantitative detection. The TDDFT-based analysis of electron distribution further validated the experimental results and enriched the theoretical understanding of the probe’s working mechanism. Overall, these results not only enhanced our knowledge of the FRET mechanism in fluorescence probes but also offer valuable theoretical inspiration for the future design of more efficient and sensitive fluorescence probes targeting similar biological parameters. However, further research could focus on improving the probe’s accuracy, reducing the deviation between calculated and experimental values, and exploring its application in more complex biological systems.

## Data Availability

The original contributions presented in the study are included in the article/Supplementary Material, further inquiries can be directed to the corresponding authors.
