# Supplementary material for: Decoding JFT: a multifunctional fluorescence probe for sulfite and viscosity insights
Source: Front Chem. 2025 Jul 23;13:1642191. doi: 10.3389/fchem.2025.1642191 (PMC12325367; doi:10.3389/fchem.2025.1642191)
Supplement: Supplementary file 1 [file DataSheet1.pdf]

Decoding JFT: A Multifunctional Fluorescence Probe for Sulfite and Viscosity Insights

Bin Han[1], Yongjin Peng[2,3*], Yuling Liu[2,3*]

1 Department of Physics, Tianjin Renai College, Tianjin 301636, P. R. China

2 College of Modern Industry of Health Management, Jinzhou Medical University, Jinzhou 121001, P. R. China

3 Liaoning Province Key Laboratory of Human Phenome Research, Jinzhou Medical University, Jinzhou 121001, P. R. China

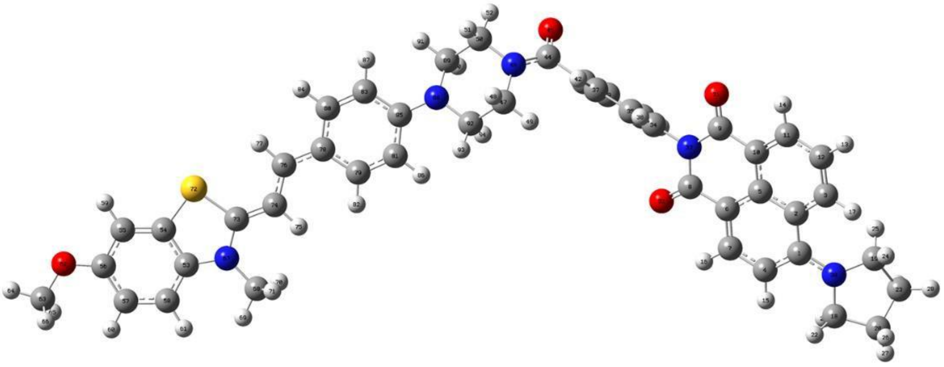

Figure S1 the structure of JFT

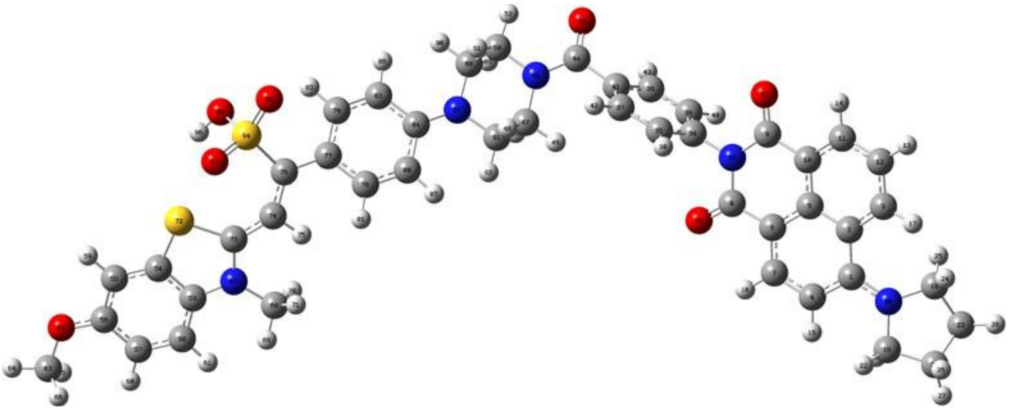

Figure S2 the structure of product of JFT reacted with sulfite

Cartesian coordinates of the probe JFT:

C	2.1361	5.37445	4.74244
C	0.82368	5.95315	4.5314
C	0.4947	7.30287	4.8081
C	2.25538	3.97705	4.65462
C	-0.2361	5.1028	4.0819
C	-0.03736	3.71252	3.92336
C	1.19521	3.17197	4.24657
C	-1.10835	2.82475	3.45492
C	-2.62769	4.79841	3.3362
C	-1.51969	5.64858	3.83279
C	-1.78035	6.98518	4.06734
C	-0.77089	7.80872	4.58304
H	-0.98437	8.85367	4.81668
H	-2.78603	7.35883	3.86489
H	3.21049	3.50056	4.86494
H	1.32398	2.09184	4.14876
H	1.23868	7.96482	5.24218
C	4.50676	5.47624	5.36834
C	3.46678	7.51772	4.65527
C	5.4879	6.62788	5.51701
H	4.83069	4.79909	4.55428
H	4.40497	4.86646	6.28062
C	4.97625	7.615	4.47726
H	3.14471	8.21063	5.45407
H	2.90617	7.77405	3.74388
H	5.41792	7.06455	6.52652
H	6.52886	6.314	5.3582

H	5.34885	8.64015	4.61234
H	5.26112	7.28251	3.46584
N	3.24455	6.12315	5.03066
O	-3.72629	5.24565	3.08946
O	-0.96692	1.62884	3.31403
N	-2.34442	3.43773	3.16483
C	-3.39379	2.59752	2.67618
C	-4.56223	2.43666	3.41754
C	-3.24608	1.94561	1.45272
C	-5.57175	1.60572	2.94441
H	-4.6826	2.97318	4.35936
C	-4.26265	1.12748	0.97747
H	-2.33022	2.08366	0.87656
C	-5.42513	0.93098	1.72812
H	-6.49208	1.49598	3.52238
H	-4.17192	0.63409	0.00799
C	-6.52101	0.10273	1.12395
O	-6.81766	0.25227	-0.04871
N	-7.17732	-0.78427	1.93784
C	-6.68434	-1.33046	3.21249
H	-7.35504	-1.07076	4.05084
H	-5.68746	-0.92988	3.42644
C	-8.328	-1.47144	1.40642
H	-9.16229	-1.43291	2.1265
H	-8.62283	-0.98819	0.46794
C	-9.99194	-12.93587	6.19239
C	-10.98064	-12.95858	5.19613
C	-11.7902	-14.06215	4.99288
C	-11.60275	-15.18321	5.81644
C	-10.61123	-15.16505	6.81743
C	-9.80538	-14.04815	7.00963
H	-12.56187	-14.09338	4.22304
H	-10.46247	-16.03328	7.45845
H	-9.04481	-14.0597	7.79123
O	-12.40866	-16.21854	5.57824
C	-12.29476	-17.3884	6.35394
H	-13.05171	-18.08438	5.97452
H	-11.29821	-17.84845	6.24659
H	-12.49431	-17.18703	7.41971
N	-9.29554	-11.72786	6.22227
C	-8.23703	-11.52625	7.19153
H	-8.64102	-11.63603	8.20812
H	-7.44283	-12.27023	7.03421
H	-7.80727	-10.52694	7.09199

S	-10.99193	-11.44582	4.32925
C	-9.69282	-10.82468	5.30479
C	-9.15285	-9.53248	5.123
H	-8.3358	-9.23117	5.77823
C	-9.61146	-8.6574	4.16643
H	-10.43562	-8.99412	3.52518
C	-9.14849	-7.34241	3.89168
C	-8.09352	-6.71194	4.59803
C	-9.76087	-6.59623	2.85498
C	-7.68079	-5.4383	4.29571
H	-7.58485	-7.24027	5.40721
C	-9.36193	-5.31979	2.53741
H	-10.57815	-7.05051	2.28838
C	-8.3021	-4.69155	3.24999
H	-6.86552	-5.0002	4.86901
H	-9.86946	-4.7943	1.73047
N	-7.89808	-3.43804	2.95028
C	-8.54249	-2.69839	1.88546
H	-8.43719	-3.20988	0.91456
H	-9.61646	-2.55101	2.08597
C	-6.8129	-2.76801	3.67174
H	-7.05197	-2.67654	4.74405
H	-5.87349	-3.33618	3.57149

Cartesian coordinates of the sulfite adduct of JFT:

C	-8.80737	-12.23344	5.73826
C	-10.16028	-12.4789	6.03198
C	-10.60281	-13.71529	6.46692
C	-9.67326	-14.75692	6.60506
C	-8.32458	-14.52716	6.30738
C	-7.88925	-13.27168	5.87468
H	-11.65065	-13.90548	6.70348
H	-7.59077	-15.32682	6.40413
H	-6.83293	-13.12959	5.64303
O	-10.17121	-15.93753	7.02572
C	-9.29907	-17.02017	7.18376
H	-9.9065	-17.8659	7.52973
H	-8.81142	-17.29801	6.23226
H	-8.51726	-16.81227	7.93603
N	-8.58438	-10.92707	5.33205
C	-7.28654	-10.43247	4.9598
H	-6.52121	-11.15216	5.26811
H	-7.20588	-10.2763	3.87068
H	-7.07618	-9.47935	5.46823
S	-11.11908	-11.02447	5.84924

C	-9.72442	-10.14246	5.21468
C	-9.67992	-8.88195	4.6356
H	-8.66723	-8.57441	4.36779
C	-10.20152	-6.62681	3.73171
C	-9.07265	-6.00356	4.29347
C	-10.82489	-5.95207	2.66048
C	-8.57773	-4.79806	3.81472
H	-8.57601	-6.46958	5.14799
C	-10.34089	-4.74733	2.18587
H	-11.71236	-6.3828	2.19935
C	-9.20332	-4.12814	2.74641
H	-7.6901	-4.37941	4.28847
H	-10.87811	-4.26329	1.36931
N	-8.75497	-2.89708	2.27824
S	-12.3477	-8.27797	4.35878
O	-12.55207	-9.56286	3.38683
H	-12.35681	-10.36668	3.90521
O	-12.61067	-8.73156	5.72092
O	-13.1464	-7.22555	3.77318
C	-10.63084	-7.93117	4.22851
H	-9.56874	-7.9526	4.10053
C	-8.75643	-2.63285	0.83218
H	-9.76396	-2.51248	0.49262
H	-8.20352	-1.73883	0.63241
H	-8.30289	-3.45491	0.31894
C	-7.66244	-2.21253	2.98441
H	-7.90668	-2.12794	4.02272
H	-6.75916	-2.77561	2.87521
H	-7.52625	-1.23578	2.56932

Cartesian coordinates of Acceptor:

C	-2.1371	-1.09715	-0.57081
C	-1.03533	-1.09129	0.30208
C	-0.33015	0.067	0.58289
C	-0.74743	1.26107	-0.02337
C	-1.85766	1.26034	-0.89313
C	-2.55308	0.08974	-1.17028
H	0.52656	0.08618	1.25735
H	-2.18705	2.18689	-1.36212
H	-3.41051	0.11728	-1.84398
O	-0.03801	2.34691	0.2834
C	-0.38553	3.5936	-0.27282
H	0.33387	4.31883	0.12432
H	-0.31086	3.57947	-1.37298
H	-1.40309	3.89811	0.02378

N	-2.67393	-2.3742	-0.732
C	-3.7816	-2.62928	-1.63235
H	-3.8031	-3.6935	-1.89102
H	-4.73598	-2.34053	-1.16733
H	-3.63713	-2.0509	-2.55404
S	-0.78133	-2.69061	0.94922
C	-2.05445	-3.33579	-0.02975
C	-2.47197	-4.69266	0.06634
H	-3.53825	-4.89271	-0.06364
C	-1.57301	-5.72759	0.1309
H	-0.50857	-5.46173	0.14011
C	-1.84693	-7.12575	0.16855
C	-3.15301	-7.67198	0.17668
C	-0.76915	-8.0408	0.21465
C	-3.36988	-9.02761	0.22501
H	-4.0222	-7.01091	0.14979
C	-0.96553	-9.40164	0.26391
H	0.25387	-7.65489	0.21351
C	-2.27952	-9.94602	0.2703
H	-4.39448	-9.39574	0.23137
H	-0.09801	-10.05815	0.30122
N	-2.48952	-11.28096	0.31864
C	-1.36774	-12.19411	0.36248
H	-1.74082	-13.22348	0.39205
H	-0.72528	-12.09176	-0.5277
H	-0.74618	-12.03267	1.25888
C	-3.83561	-11.81197	0.33061
H	-3.79135	-12.9055	0.37448
H	-4.40451	-11.4613	1.20761
H	-4.39251	-11.53244	-0.57909

Cartesian coordinates of Donor:

C	1.70646	4.55052	5.5525
C	0.70181	5.25305	4.77836
C	0.66589	6.65903	4.60992
C	1.4825	3.1917	5.83303
C	-0.36846	4.49949	4.19967
C	-0.49677	3.11271	4.44087
C	0.41189	2.49322	5.28167
C	-1.58124	2.32034	3.84983
C	-2.44507	4.39637	2.77024
C	-1.34366	5.15521	3.40812
C	-1.30777	6.5248	3.2274
C	-0.30981	7.28134	3.85554
H	-0.30501	8.36869	3.75704

H	-2.08371	6.98567	2.61299
H	2.18301	2.64563	6.46117
H	0.28127	1.42789	5.48367
H	1.40017	7.2844	5.10974
C	3.7469	4.4314	6.91056
C	3.51045	6.31258	5.43965
C	4.90757	5.39102	7.11824
H	4.10209	3.49711	6.43409
H	3.23853	4.14914	7.84667
C	4.98101	6.09457	5.77034
H	3.1649	7.25573	5.90111
H	3.31489	6.3773	4.35886
H	4.6704	6.11433	7.91526
H	5.83412	4.8722	7.4004
H	5.55053	7.03444	5.7868
H	5.44395	5.432	5.02111
N	2.84162	5.15165	6.02298
O	-3.27572	4.92761	2.06636
O	-1.71825	1.13087	4.04216
N	-2.48435	3.01899	3.02241
C	-3.53216	2.26249	2.41059
C	-4.86319	2.53728	2.71834
C	-3.21763	1.25414	1.50067
C	-5.87655	1.78699	2.13268
H	-5.10217	3.34763	3.40787
C	-4.23339	0.51508	0.90817
H	-2.17284	1.05245	1.2611
C	-5.56986	0.75882	1.23525
H	-6.9197	2.01575	2.36318
H	-4.00248	-0.25942	0.17433
C	-6.62709	-0.02233	0.51547
O	-6.54351	-0.20989	-0.68534
N	-7.6922	-0.47543	1.25376
C	-7.68493	-0.72898	2.67743
H	-8.58291	-0.27375	3.13206
H	-6.80115	-0.27399	3.13889
C	-8.776	-1.14474	0.56697
H	-9.73293	-0.71657	0.9134
H	-8.66001	-0.95895	-0.50774
